# Pro-inflammatory cerebrospinal fluid profile of neonates with intraventricular hemorrhage: clinical relevance and contrast with CNS infection

**DOI:** 10.1186/s12987-024-00512-0

**Published:** 2024-02-21

**Authors:** Maria Garcia-Bonilla, Alexander T. Yahanda, Albert M. Isaacs, Brandon Baksh, S. Hassan A. Akbari, Haley Botteron, Diego M. Morales, Rowland H. Han, James P. McAllister II, Amit M. Mathur, Jennifer M. Strahle, Christopher D. Smyser, David D. Limbrick

**Affiliations:** 1 Department of Neurosurgery, Washington University School of Medicine, One Children’s Place, Suite 420, St. Louis, MO USA; 2 Department of Neurosurgery, Virginia Commonwealth University School of Medicine, Richmond, VA USA; 3 Department of Neurosurgery, Nationwide Children’s Hospital, Ohio State University, Columbus, OH USA; 4 University of Miami Miller School of Medicine, Miami, FL USA; 5 John Hopkins University School of Medicine, Johns Hopkins All Children’s Hospital, St. Petersburg,, FL USA; 6 Medical School, University of Kansas, Kansas City, KS USA; 7 Department of Pediatrics, Saint Louis University School of Medicine, St. Louis, MO USA; 8 Department of Pediatrics, Washington University School of Medicine, St. Louis, MO USA; 9 Department of Neurology, Washington University School of Medicine, St. Louis, MO USA; 10 Mallinckrodt Institute of Radiology, Washington University School of Medicine, St. Louis, MO USA

**Keywords:** Bacterial Meningitis, Cerebrospinal fluid, CNS infection, Intraventricular hemorrhage, Prematurity, Post-hemorrhagic hydrocephalus, Viral meningitis

## Abstract

**Background:**

Interpretation of cerebrospinal fluid (CSF) studies can be challenging in preterm infants. We hypothesized that intraventricular hemorrhage (IVH), post-hemorrhagic hydrocephalus (PHH), and infection (meningitis) promote pro-inflammatory CSF conditions reflected in CSF parameters.

**Methods:**

Biochemical and cytological profiles of lumbar CSF and peripheral blood samples were analyzed for 81 control, 29 IVH grade 1/2 (IVH_1/2_), 13 IVH grade 3/4 (IVH_3/4_), 15 PHH, 20 culture-confirmed bacterial meningitis (BM), and 27 viral meningitis (VM) infants at 36.5 ± 4 weeks estimated gestational age.

**Results:**

PHH infants had higher (*p* < 0.02) CSF total cell and red blood cell (RBC) counts compared to control, IVH_1/2_, BM, and VM infants. No differences in white blood cell (WBC) count were found between IVH_3/4_, PHH, BM, and VM infants. CSF neutrophil counts increased (*p* ≤ 0.03) for all groups compared to controls except IVH_1/2_. CSF protein levels were higher (*p* ≤ 0.02) and CSF glucose levels were lower (*p* ≤ 0.003) for PHH infants compared to all other groups. In peripheral blood, PHH infants had higher (*p* ≤ 0.001) WBC counts and lower (*p* ≤ 0.03) hemoglobin and hematocrit than all groups except for IVH_3/4_.

**Conclusions:**

Similarities in CSF parameters may reflect common pathological processes in the inflammatory response and show the complexity associated with interpreting CSF profiles, especially in PHH and meningitis/ventriculitis.

**Supplementary Information:**

The online version contains supplementary material available at 10.1186/s12987-024-00512-0.

## Introduction

Analysis of cerebrospinal fluid (CSF) is an essential component of the evaluation and treatment of newborn neurological and infectious disorders. Classically, the CSF profile of infectious meningitis, for example, comprises pleocytosis with a preponderance of white blood cells (WBC), elevated protein levels, and low glucose levels [1, 2]. However, CSF differential cell count and biochemical profile may be influenced by a myriad of factors, all of which may occur on a systemic inflammatory background associated with preterm birth and/or comorbid conditions [3, 4].

Intraventricular hemorrhage (IVH), and resultant post-hemorrhagic hydrocephalus (PHH), are among the most common, severe neurological complications of preterm birth, and infants frequently require CSF sampling for diagnostic or therapeutic purposes [5, 6]. Growing evidence suggests that the antecedent IVH and PHH are associated with host-immune responses that can alter the CSF profile [7–12], potentially prompting empiric antimicrobial therapy on suspicion of infection or delaying time-sensitive surgical care [13, 14].

We hypothesized that IVH, PHH, and meningitis promote pro-inflammatory CSF states that are reflected in CSF/peripheral blood parameters and show common pathological inflammatory processes. We compared the CSF and peripheral blood of infants with viral (VM) and bacterial (BM) meningitis to infants with IVH or PHH (without infection) and control infants.

## Methods

### Patient selection and sample collection

CSF and peripheral blood profiles of 6 groups of infants at an estimated gestational age (EGA) of 36.5 ± 4 weeks were assessed: 1) Control: no known neurological disease who required CSF sampling for routine sepsis evaluation but were found to have sterile CSF; 2) IVH_1/2_: Papile Grade 1/2 IVH identified on cranial ultrasound but no hydrocephalus [15, 16] who had sterile CSF; 3) IVH_3/4_: Papile Grade 3/4 IVH without hydrocephalus [15, 16] who had sterile CSF; 4) PHH: hydrocephalic infants who required neurosurgical intervention and had sterile CSF, sample collected prior to surgery; 5) BM: culture-confirmed bacterial meningitis with growth of aerobic bacteria in CSF culture but with negative viral RNA on PCR and no IVH/PHH; and 6) VM: PCR-confirmed viral meningitis with negative bacterial cultures and no IVH/PHH. Well-established Hydrocephalus Clinical Research Network criteria were applied to identify the severity of the hemorrhage by ultrasound to determine IVH grade (1/2 or 3/4), the diagnosis of PHH, and neurosurgical intervention [5, 17]. Clinical signs and symptoms such as fever, poor feeding, vomiting, lethargy, and irritability were also evaluated [18–21], but lumbar puncture results were essential for establishing meningitis diagnosis [22].

All samples were acquired in the Neonatal Intensive Care Unit at St. Louis Children’s Hospital (October 2006-December 2016). CSF samples for all groups were obtained via lumbar puncture (LP) and analyzed at equivalent time points (Table 1). Peripheral blood samples were collected and analyzed within 0–72 h from the CSF sample. CSF microbiological cultures were monitored for 3.68 ± 0.13 days for bacterial growth [23]. Anaerobic CSF cultures were not routinely performed.

**Table 1:** Subject characteristic for those enrolled in study

	Control (n = 81)	IVH_1/2_ (n = 29)	IVH_3/4_ (n = 13)	PHH (n = 15)	BM (n = 20)	VM (n = 27)	All patients (n = 185)
Birth EGA (weeks)	37.1 ± 3.3	33.4 ± 5.3	27.6 ± 5.7	26.6 ± 3.1	36.6 ± 4.5	37.2 ± 3	35 ± 5.3
Sample EGA (weeks)	40.4 ± 2.2	35.8 ± 4.6	32.4 ± 6.1	29.4 ± 2.8	38.9 ± 4.3	40.8 ± 1.7	38. ± 4.9
Gender (% male)	54.3	69.0	61.5	73.3	45.0	59.2	58.4
Postnatal steroids (%)	2.5	17.2	0.00	25.0	5.0	0.0	8.1
PROM (%)	9.3	35	23	40	15.38	4.34	13.51
Chorioamnionitis (%)	7.4	10.34	23.07	6.66	5	0	7.56
Lung disease (%)	9.87	27.58	23.07	73.33	35	0	20
Sepsis (%)	3.75	34.48	46.15	33.33	20	0	15.13

Data shown as mean ± standard deviation

Birth EGA statistically significant comparisons: control vs IVH_1/2_* p* = 0.0015, vs IVH_3/4_ *p* < 0.0001, vs PHH *p* < 0.0001; IVH_1/2_ vs IVH_3/4_ *p* < 0.0001, vs PHH *p* < 0.0001, vs VM *p* = 0.0154; IVH_3/4_ vs BM *p* < 0.0001, vs VM *p* < 0.0001; PHH vs BM *p* < 0.0001, vs VM *p* < 0.0001. Sample EGA statistically significant comparison: control vs IVH_1/2_ *p* < 0.0001, vs IVH_3/4_ *p* < 0.0001, vs PHH *p* < 0.0001; IVH_1/2_ vs IVH_3/4_ *p *= 0.0308, vs PHH *p* < 0.0001, vs BM *p* = 0.0184, vs VM *p* < 0.0001; IVH_3/4_ vs BM *p* = 0.0004, vs VM *p* < 0.0001; PHH vs BM *p* < 0.0001, vs VM *p* < 0.0001. PROM was statistically different between control vs IVH_1/2_ *p *= 0.011, control vs PHH* p* = 0.008, IVH_1/2_ vs VM *p *= 0.0165, and PHH vs VM *p *= 0.0096. The % of patients with chorioamnionitis was similar except of IVH_3/4_ vs VM p = 0.0289. Lung disease: control vs IVH_1/2_ *p *= 0.0306, control vs PHH *p* < 0.0001, control vs BM *p *= 0.0101, IVH_1/2_ vs PHH *p* = 0.0089; IVH_1/2_ vs BM *p* = 0.0046; IVH_3/4_ vs PHH *p* = 0.0213; IVH_3/4_ vs VM *p* = 0.0289; PHH vs BM *p* = 0.0409, PHH vs VM *p* < 0.0001, BM vs VM* p* = 0.0012. Sepsis: control vs IVH_1/2_* p* < 0.0001, vs IVH_3/4_ *p* = 0.0002, vs PHH* p* = 0.0022, vs BM *p* = 0.028; IVH_1/2_ vs VM *p* = 0.0009; IVH_3/4_ vs VM *p* = 0.0005; PHH vs VM *p* = 0.004; BM vs VM *p* = 0.0297

### Data collection

CSF parameters were retrieved from the medical records: total cell counts, red blood cell (RBC) count, WBC counts (neutrophil, lymphocyte, monocyte, eosinophil, and macrophage counts), protein, glucose, microbial cultures, and PCR results. Control LP samples with RBC counts greater than 10,000 cells/µl but no xanthochromia were excluded to minimize the effects of traumatic sampling [24]. Peripheral blood sample profile included glucose, hemoglobin, hematocrit, WBC (including neutrophils), RBC, and platelets counts. The CSF/blood glucose ratio was calculated by dividing the CSF levels between the blood levels collected within 2 days of the CSF samples. Additional data recorded and analyzed included EGA at birth and sample collection, gender, postnatal steroids, premature rupture of the membrane (PROM), chorioamnionitis, lung disease [pneumothorax, pulmonary hemorrhage, pulmonary interstitial emphysema, persistent pulmonary hypertension of the newborn (PPHN), chronic lung disease, respiratory distress syndrome (RDS), and bronchopulmonary dysplasia (BPD)], sepsis, and clinical risk index for babies (CRIB) [25, 26] scores. Patients missing the data necessary to determine the study outcomes were not included.

### Statistical analysis

Analyses were performed in STATA version 16.1 (StataCorp, College Station, TX) and GraphPad Software version 9.2.0 (San Diego, CA, USA). Groups were analyzed with Analysis of Variance (ANOVA) with Tukey–Kramer adjustment to correct for multiple pairwise comparisons. Variance components for each group were estimated to control for unequal variance when necessary. Fisher’s exact test was applied to compare categorical data. CRIB scores, PROM, lung disease, and steroid treatment were collected in preterm subjects and used for exploratory analyses through simple linear regressions [27, 28]. A two-sided *p* < 0.05 was considered statistical significant. Groups were blinded for all analyses.

## Results

### Patient Characteristics

CSF profile data from 185 infants were analyzed, 77 (41.6%) females and 108 (58.4%) males: 81 controls, 29 IVH_1/2_, 13 IVH_3/4_, 15 PHH, 20 culture-confirmed BM, and 27 PCR-confirmed VM. Post-menstrual age (PMA) at birth was 34.9 ± 5.3 weeks, and EGA at sampling was 36.5 ± 4 weeks (Table 1). Postnatal steroids were administered in 15 (8.1%) patients. PROM was more common in PHH vs. control and VM groups, but the percentage of patients with chorioamnionitis was similar among groups except for IVH_3/4_ vs. VM. Patients with lung disease were more frequent in PHH compared to all other groups. Sepsis was more frequent in all the other groups compared to control and VM groups (Table 1). In PHH, PROM, lung disease, and steroid treatment did not correlate with CSF and blood parameters except for steroid treatment and hemoglobin (R [2] = 0.3 *p* = 0.039). The exploratory analyses of CRIB scores across IVH_1/2_, IVH_3/4_, and PHH groups showed no consistent correlations with CSF/peripheral blood parameters (Additional file 1: Table S1). No correlations between CRIB scores and CSF and serum parameters were found except for CSF lymphocytes (R [2] = 0.3,* p* = 0.03), serum red blood cells (R [2] = 0.3, *p* = 0.039), hemoglobin (R [2] = 0.46,* p* = 0.0075), and hematocrit (R [2] = 0.38,* p* = 0.018) in PHH; CSF protein levels (R [2] = 0.43, *p* = 0.0001) in IVH_1/2_; serum platelets (R [2] = 0.71,* p* = 0.001) in IVH_3/4_; and estimated gestational age at birth in PHH (R [2] = 0.43, *p* = 0.0081) and both IVHs (R [2] = 0.76,* p* < 0.0001; R [2] = 0.81,* p* < 0.0001).

The most common organism present in the CSF of BM samples was coagulase-negative *Staphylococcus* in 7 (35%) patients. For VM samples, the most common virus was *Enterovirus* in 21 (78%) patients (Additional file 2: Table S2). 67 (38%) of total patients had received antibiotics within 0.5 ± 0.9 days prior to their CSF collection but subgroup analyses did not reveal any associations between antibiotics and CSF profile, except for peripheral blood platelets.

### CSF Cell counts

**Fig. 1:**
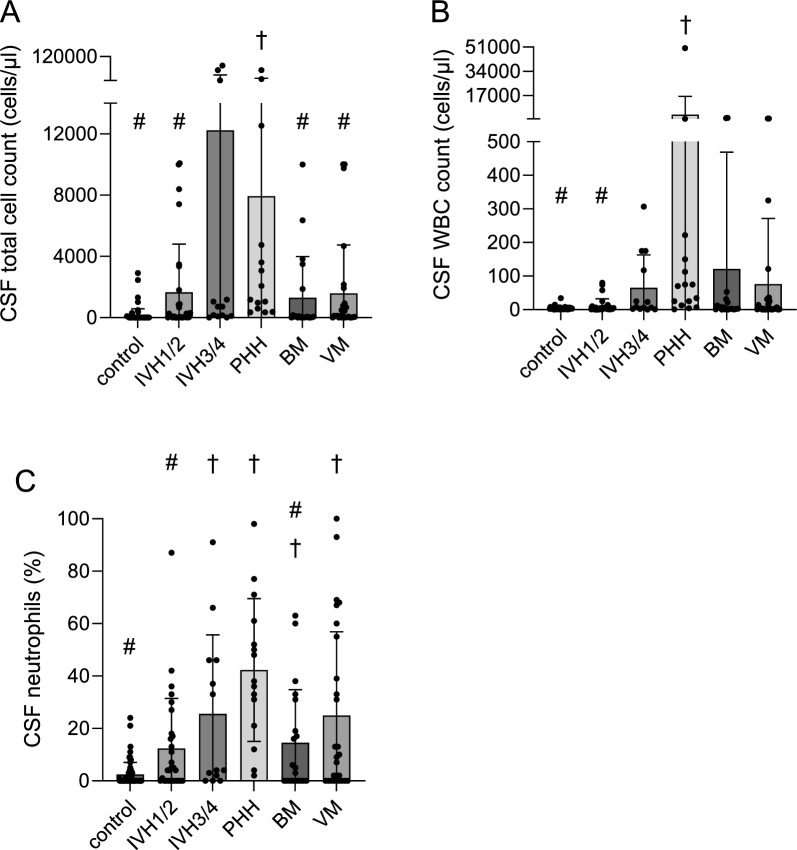
Cerebrospinal fluid total cell, white blood cell, and neutrophil counts profile. **A** Cerebrospinal fluid total cell count, **B** white blood cell (WBC) count, and **C** the percentage of neutrophils variations across groups. The # denotes the difference with the PHH group. The † denotes the difference with the control group. For specific *p*-values, see Table 3. BM, bacterial meningitis; IVH, intraventricular hemorrhage; PHH, post-hemorrhagic hydrocephalus; VM, viral meningitis

PHH group had significantly higher (*p* < 0.02) total cell counts (7925 ± 16,016 cells/µl), including RBC counts (7829 ± 16,017 cells/µl), compared to all other groups except for IVH_3/4_. There were no differences in total cells, WBC, or RBC counts between the IVH_1/2_, IVH_3/4_, BM and VM groups (Fig. 1; Tables 2 and 3). There was no difference in total cells, RBCs, WBCs, when comparing BM and VM organism subcategories (Additional file 3: Table S3).

**Table 2:** CSF and peripheral blood profiles of patients

	Control	IVH_1/2_	IVH_3/4_	PHH	BM	VM
CSF						
Protein (mg/dL)	65.44 ± 27.01^#^	109.84 ± 47.49^#†^	199.72 ± 87.69^#†^	286.7 ± 156.2^†^	132.52 ± 120.1^#†^	78.93 ± 51.33^†^
Glucose (mg/dL)	52.67 ± 11.41^#^	50.07 ± 11.31^#^	48.27 ± 26.97^#^	32.11 ± 9.32^†^	53 ± 14.26^#^	53.15 ± 11.06^#^
Total Cells (cells/μL)	125.5 ± 454.1^#^	1661.34 ± 3136^#^	12,224.85 ± 26,345	7925.36 ± 16,016^†^	1308.1 ± 2681^#^	1586.67 ± 3165^#^
WBC (cells/μL)	3.46 ± 4.30^#^	11.21 ± 21.39^#^	65.15 ± 98.02	3439 ± 12,950^†^	121.20 ± 348.1	76.37 ± 195.4
RBC (cells/μL)	122 ± 454.4^#^	1650.14 ± 3123^#^	12,159 ± 26,276	7829.71 ± 16,017^†^	1186.9 ± 2641^#^	1510.3 ± 3188^#^
Neutrophils (%)	2.44 ± 4.59^#^	12.41 ± 19.07^#^	25.54 ± 30.13^†^	42.27 ± 27.26^#†^	14.55 ± 20.27^#†^	24.92 ± 31.94^†^
Lymphocytes (%)	28.83 ± 23.47	28.41 ± 17.41	17.77 ± 12.42	15.93 ± 12.56	26.5 ± 19.75	23.63 ± 17.35
Monocytes (%)	60.37 ± 25.55^#^	50.03 ± 26.75	36.86 ± 28.41^†^	29.60 ± 24.67^†^	49.95 ± 26.78	44.63 ± 30.12
Eosinophils (%)	0.02 ± 0.22	2.07 ± 3.88^†^	0.54 ± 0.97	1 ± 0.88	0.75 ± 1.59	0.0 ± 0.0
Macrophages (%)	5.93 ± 11.8	5.07 ± 7.57	17.15 ± 27.51^†^	8.43 ± 13.47	3.90 ± 8.63	4.26 ± 11.1
Peripheral blood						
WBC (K/μL)	11.07 ± 5.45^#^	13.18 ± 8.12^#^	15.70 ± 10.38	22.26 ± 7.83^†^	12.39 ± 8.39^#^	10.63 ± 4.39^#^
RBC (K/μL)	3.84 ± 0.79	3.93 ± 0.77	3.47 ± 0.7	3.36 ± 0.48	3.88 ± 0.83^#^	4.09 ± 0.76
Hemoglobin (g/dL)	12.90 ± 3^#^	13.37 ± 3.37^#^	10.65 ± 3.09	10.08 ± 1.93^†^	13.06 ± 2.85^#^	13.74 ± 3.02^#^
Hematocrit (%)	37.35 ± 8.34^#^	38.80 ± 9.16^#^	31.16 ± 8.41	29.82 ± 5.54^†^	37.96 ± 7.87^#^	40.15 ± 8.39^#^
Platelets (K/μL)	387.90 ± 355	251.17 ± 118.4	190.72 ± 131.1	247.29 ± 91.2	300.93 ± 125.2	325.20 ± 150
Neutrophils^#^	0.98 ± 4.7	6.78 ± 18.59^†^	3.77 ± 4.5	1.61 ± 2.41	3.28 ± 6.34	0.04 ± 0.2

Data shown as mean ± standard deviation. The # denotes difference with the PHH group

The † denotes difference with the control group. For specific *p*-values, see Table 3

**Table 3:** *P*-values for groupwise comparisons of all groups

	BM: Control	BM: IVH_1/2_	BM: IVH_3/4_	BM: PHH	BM: VM	Control:IVH_1/2_	Control: IVH_3/4_	Control: PHH	Control: VM	IVH_1/2_: IVH_3/4_	IVH_1/2_: PHH	IVH_3/4_: PHH	IVH_1/2_: VM	IVH_3/4_: VM	PHH: VM
CSF															
Protein	0.005*	0.98	0.016*	< 0.001*	0.20	0.045*	< 0.001*	< 0.001*	0.99	0.002*	< 0.001*	0.019*	0.51	< 0.001*	< 0.001*
Glucose	1.00	0.99	0.99	0.001*	0.99	0.95	0.99	0.0002*	0.99	0.95	0.003*	0.001*	0.88	1.00	< 0.001*
Total Cells	1.00	1.00	0.87	0.009*	1.00	1.00	0.71	0.001*	1.00	0.88	0.008*	0.39	1.00	0.9	0.018*
WBC	1.00	1.00	1.00	0.056	1.00	1.00	1.00	0.012*	1.00	1.00	0.039*	0.14	1.00	1.00	0.073
RBC	1.00	1.00	0.79	0.007*	1.00	1.00	0.59	0.001*	1.00	0.80	0.007*	0.44	1.00	0.84	0.015*
Neutrophils	0.03*	0.99	0.63	< 0.001*	0.28	0.17	0.001*	< 0.001*	< 0.001*	0.33	< 0.001*	0.21	0.09	1.00	0.22
Lymphocytes	0.99	0.99	0.79	0.57	0.99	1.00	0.44	0.20	0.81	0.60	0.37	1.00	0.92	0.98	0.93
Monocytes	0.41	1.00	0.75	0.21	0.99	0.47	0.042*	0.001*	0.14	0.68	0.16	0.98	0.97	0.98	0.62
Eosinophils	0.71	0.011*	1.00	0.97	0.87	< 0.001*	0.91	0.34	1.00	0.072	0.37	0.98	< 0.001*	0.95	0.52
Macrophages	0.99	1.00	0.05*	0.95	0.99	1.00	0.041*	0.98	0.96	0.055	0.97	0.48	0.99	0.029*	0.85
Blood															
WBC	1.00	0.93	0.55	0.001*	1.00	0.79	0.37	< 0.001*	1.00	0.94	0.001*	0.08	0.88	0.49	< 0.001*
RBC	0.68	0.98	0.18	0.032*	0.99	0.99	0.65	0.21	0.97	0.48	0.14	0.99	1.00	0.47	0.16
Hemoglobin	0.70	0.99	0.03*	0.001*	0.98	0.97	0.17	0.008*	0.99	0.1	0.006*	0.98	1.00	0.21	0.022*
Hematocrit	0.64	0.99	0.024*	0.001*	0.62	0.96	0.16	0.01*	0.99	0.08	0.006*	0.99	1.00	0.15	0.018*
Platelets	0.87	0.85	0.81	0.98	0.99	0.1	0.21	0.34	0.72	0.99	1.00	1.00	0.98	0.95	0.99
Neutrophils	0.99	0.29	0.99	1.00	0.96	0.019*	0.91	1.00	0.99	0.84	0.37	0.99	0.07	0.86	0.99

The bold emphasis and * denote statistical significance

The PHH group had significantly higher mean WBC counts than the control (*p* = 0.012) and IVH_1/2_ (*p* = 0.039) groups (Fig. 1; Tables 2 and 3). On WBC differential analyses, neutrophil counts of all groups significantly differed from controls except for IVH_1/2_ (*p* ≤ 0.05). The PHH cohort had significantly higher neutrophil counts than every group save for the VM and IVH_3/4_ groups. WBC counts were not different between infants with CNS infections. The remaining nucleated cell lines did not demonstrate consistent differences between groups except for eosinophils in IVH_1/2_ compared to control (*p* < 0.001), BM (*p* = 0.01), and VM (*p* < 0.001; Fig. 1; Tables 2 and 3).

### CSF *protein levels*

**Fig. 2:**
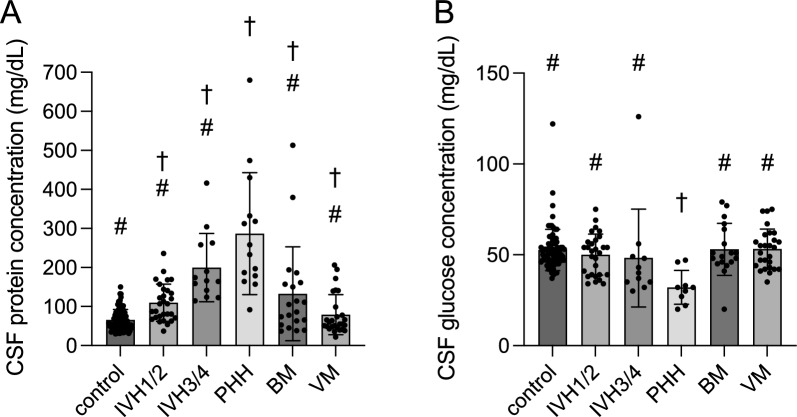
Cerebrospinal fluid protein and glucose concentrations. **A** Cerebrospinal fluid protein, and **B** glucose concentrations. The # denotes the difference with the PHH group. The † denotes the difference with the control group. For specific *p*-values, see Table 3. BM, bacterial meningitis; IVH, intraventricular hemorrhage; PHH, post-hemorrhagic hydrocephalus; VM, viral meningitis

Protein levels in the PHH group were significantly higher than that of all groups (*p* ≤ 0.001; Fig. 2A; Tables 2 and 3). There was no difference between the BM and VM groups, although the BM group significantly differed from controls (*p* = 0.005). The IVH_1/2_ group had higher protein levels than the control group (*p* = 0.045) but did not differ from the BM group. However, the IVH_3/4_ group had significantly higher protein levels than the VM group (*p* < 0.001; Fig. 2A; Tables 2 and 3).

### CSF glucose levels

CSF glucose levels were significantly lower in the PHH group than all other groups (*p* ≤ 0.003; Fig. 2B; Tables 2 and 3). There were no differences between BM and VM groups, and these groups did not differ from the control group. Neither IVH group differed from any other group besides the PHH group (*p* ≤ 0.003) (Fig. 2B; Tables 2 and 3). Furthermore, CSF/blood glucose ratios were significantly lower (*p* < 0.004) in the PHH (0.34 ± 0.14) and IVH_3/4_ (0.38 ± 0.13) groups compared to the control group (0.65 ± 0.26), and the PHH glucose ratio was lower (*p* = 0.0406) than VM (0.63 ± 0.15).

### Peripheral blood biochemical and cellular profiles

**Fig. 3:**
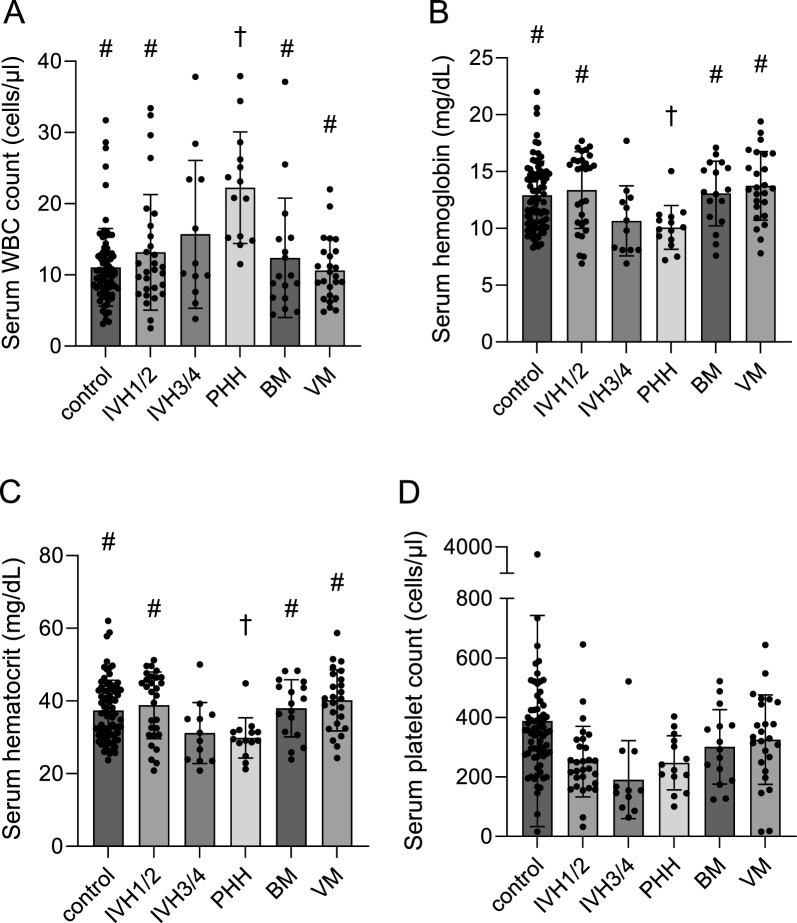
Peripheral blood white blood cells, hemoglobin, hematocrit, and platelets profile.** A** Peripheral blood white blood cell (WBC) counts, **B** hemoglobin, **C** hematocrit, and **D** platelet counts across groups. The # denotes the difference with the PHH group. The † denotes the difference with the control group. For specific *p*-values, see Table 3. BM, bacterial meningitis; IVH, intraventricular hemorrhage; PHH, post-hemorrhagic hydrocephalus; VM, viral meningitis

The PHH group had significantly higher peripheral blood WBC cell counts compared to control (*p* < 0.001), BM (*p* = 0.001), VM (*p* < 0.001), and IVH_1/2_ (*p* = 0.001) groups (Fig. 3; Tables 2 and 3). There were no differences between the non-PHH groups. In contrast, PHH infants had lower hemoglobin and hematocrit levels compared to IVH_1/2_ (*p* = 0.006), VM (*p* = 0.022 and *p* = 0.018), BM (*p* = 0.001) and control (*p* = 0.008 and *p* = 0.01) groups. There were no differences between the non-PHH groups with the exception of the IVH_3/4_ and BM groups (*p* = 0.024; Fig. 3; Tables 2 and 3).

## Discussion

This study characterizes the spectrum of CSF biochemical and cellular profiles in PHH and non-PHH infants both with and without CNS infections. PHH CSF had the highest cell count and protein levels and the lowest glucose levels among all infants. PHH group had higher peripheral blood WBCs, lower hemoglobin and lower percent hematocrit than control, IVH_1/2_, BM, and VM groups, and higher peripheral blood WBCs compared to BM and VM groups. These similarities in CSF and peripheral blood counts suggest common pathological inflammatory processes among groups and reflex the complexity in interpretating CSF profiles.

PHH and BM demonstrated similar patterns of biochemical changes when compared to controls suggesting no set thresholds to distinguish infected from non-infected samples. While we assessed for the role of WBC as a complement to facilitate such decisions, we did not identify any reliable trends. We did identify a higher peripheral blood WBC counts in PHH infants compared to controls, IVH_1/2_, VM, and BM infants, but this may be an artifact of co-morbid conditions and/or prior infection and should be interpreted with caution. PHH infants may be more seriously ill than non-PHH infants, which also likely yields higher peripheral WBC counts.

Differentiating PHH and BM CSF findings is an important clinical challenge, as CSF profiles are often used as a basis for initiating empiric antimicrobial therapy or deferring intervention when CNS infection is suspected in a PHH infant [29]. While the consequences of untreated CNS infection can be devastating, preemptive therapies are not without risks [30]; therefore, caution should be exercised in the use of empiric antibiotic treatment in PHH based on their CSF profiles alone, as the CSF altered profile is likely simply reflective of PHH pathophysiology. Treatment recommendations would change, of course, if there were other clinical factors to suggest an infection such as sustained fevers, altered level of consciousness, identified extracranial source of infection, or sepsis. Uncovering new biomarkers for diagnosing meningitis and determining antibiotic prescription among preterm infants is needed and will help to differentiate infections versus other diseases and the timing of the treatment [31].

Classically, BM is characterized by elevated CSF protein, WBCs and decreased CSF glucose [32]. Elevated WBCs have been attributed to the effects of the inflammatory response to infection [33]. Cytokines and chemokines are produced and trigger an inflammatory cascade including interleukin-1 (IL-1), which increases the permeability of the blood–brain barrier (BBB) [34]. Greater BBB permeability permits proteins from systemic circulation to enter the subarachnoid space, raising CSF protein levels. Pro-inflammatory mediators such as tumor necrosis factor alpha (TNF-α) and IL-1 also correlate with the production of nitric oxide metabolites, which decrease oxygen uptake and yield increased CSF lactate and decreased CSF glucose levels through anaerobic glycolysis [33–35].

The VM group was comprised of subjects infected by four different viruses, the most common of which was enterovirus. The sequelae of VM are typically similar to that of BM, albeit less severe. Once the virus crosses the BBB it also triggers a response from lymphocytes and inflammatory cytokines [36]. Because the inflammatory response differs depending on the infecting pathogen, the CSF profile characteristic of a viral infection is variable, while the CSF usually demonstrates elevated WBC counts and protein levels [36]. Also, a compromised BBB may result in increased CSF protein levels as well as elevated total cell count [37].Elevated WBC counts and protein levels were seen in the VM cohort, which had increased WBC counts but only moderately higher protein levels from controls. However, unlike the BM cohort, glucose in the VM cohort remained around the normal level. Moving forward, insight into inflammatory processes may be provided through the addition of CSF markers such as cytokines and chemokines levels to CSF cell profiling.

Few previous studies have investigated CSF parameters in children with PHH [38, 39]. The protein and glucose levels identified in our PHH group were close to the range of the levels reported by both of the aforementioned studies. The mean CSF WBC count in our PHH group, exclusively obtained via LP, was more than they reported. Regarding these notable differences, the authors posited that large increases in CSF cell counts and proteins may be attributed to insertions of reservoirs, as significant decreases in CSF protein and neutrophils were observed over serial samples. Our study analyzed CSF samples prior to reservoir insertion suggesting that these alterations might not be related to ventricular diversion but from broader inflammatory process. Additionally, our study adds new comparisons to previous literature as it includes blood parameters and multiple comparison groups to assess if infection could be discerned in the absence of a positive culture. We also included CSF/blood glucose ratio as a helpful measurement for differential diagnosis [40, 41]. Thus, the increased CSF and peripheral blood cell counts detected in PHH infants might be associated with early inflammatory processes inherent to the disease and not related to device insertion.

Exacerbated alterations of CSF composition in PHH is suggestive of the activation of common inflammatory pathways in the PHH and BM groups. Recently, studies have demonstrated strong associations between CSF inflammatory markers and PHH including IL-1β, IL-6, and TNF-α [8–12]. Additionally, Karimy et al. [42] found that IVH caused CSF hypersecretion in an inflammatory-dependent manner involving TLR4 and NF-κB signaling. Similar inflammatory markers have been demonstrated in the pathophysiology of BM [33]. CSF IL-6 and IL-10 were found to be strong predictors of culture-proven BM [35]. Together, these data strengthen the argument that PHH and BM involve similar inflammatory processes [43]. Supporting this hypothesis, PHH together with IVH, showed the highest number of patients with PROM, lung disease, and sepsis. PROM is associated with several neonatal diseases, and it is one of the major risk factors for neonatal sepsis [44], and lung diseases [45]. The pro-inflammatory molecules IL-6, IL-8, and TNF-α, which are increased in PHH, have also been associated with neonatal sepsis with PROM [46]. However, whether inflammation drives development of PHH or vice-versa is unclear and requires additional investigation. It is important to note that the higher CSF total cells in the PHH group compared to the BM group was likely driven by far higher levels of RBCs in the PHH group.

Limitations of this study include differences between study groups: PMA at birth and EGA at sampling, use of antibiotics, comorbid illnesses, and timing of sample collection. Although many of our subjects received empirically administered antibiotics prior to sample procurement, our analysis on the effect of antibiotics demonstrated no major differences between those treated and not treated with antibiotics. Data in the literature on the effect of antibiotics in CSF parameters are inconclusive. Srinivasan et al. [47] found antibiotics had no effect on CSF parameters, while Nigrovic et al. [48] found significant effects on CSF protein and glucose. Additional limitations of the study include its retrospective nature and the small sample sizes in certain groups. Further studies with larger samples obtained prospectively, as well as a comparative cohort study of PHH infants with culture-proven infection, would further delineate the differences in CSF profiles. Finally, systematic data on antenatal steroid use and hypertension were missing, which could have altered inflammatory factors in CSF after birth.

In conclusion, CSF profiles of patients with PHH included elevated CSF protein, total cells, RBCs, and WBCs (neutrophils and monocytes) with decreased glucose levels compared to healthy values. The PHH CSF profile was similar to that of BM or VM when compared to controls, and may be more extreme in terms of protein, glucose, RBC, and total cell counts. There was variation in PHH blood parameters compared with control, VM, and BM groups, with higher peripheral blood WBC count and lower hemoglobin and percent hematocrit. Infants with IVH had similar CSF profiles to PHH but less exacerbated alterations. The similarities in CSF among these groups may reflect common pathological processes in the inflammatory response. Therefore, CSF profile alone should not dictate the administration of empiric antimicrobial therapy in preterm IVH/PHH neonates until CNS infection is culture-proven or supported by high clinical suspicion.

### Supplementary Information

**Additional file 1: Table S1.** Percentage of preterm neonates in the CRIB score categories.

**Additional file 2: Table S2.** Bacterial and viral organisms identified in the cerebrospinal fluid of infants with meningitis.

**Additional file 3: Table S3.** Cell counts by organism for bacterial and viral meningitis groups.

## Data Availability

Data are available upon request.
