# Supplementary material for: Pro-inflammatory cerebrospinal fluid profile of neonates with intraventricular hemorrhage: clinical relevance and contrast with CNS infection
Source: Fluids Barriers CNS. 2024 Feb 21;21:17. doi: 10.1186/s12987-024-00512-0 (PMC10880312; doi:10.1186/s12987-024-00512-0)
Supplement: Supplementary file 1 — Additional file 1: Table S1. Percentage of preterm neonates in the CRIB score categories. [file 12987_2024_512_MOESM1_ESM.docx]

| Additional file 1: Table S1. Percentage of preterm neonates in the CRIB score categories. | | | |
| --- | --- | --- | --- |
| CRIB scores | **IVH_1/2_**  **(n=29)** | **IVH_3/4_**  **(n=13)** | **PHH**  **(n=15)** |
| 0 | 31.03 % | 15.38 % | 0 % |
| 1 | 24.13 % | 7.69 % | 13.33 % |
| 2 | 17.24 % | 15.38 % | 26.66 % |
| 3 | 20.68 % | 38.46 % | 40 % |
| 4 | 6.89 % | 23.07 | 20 % |
| Abbreviations: CRIB, clinical risk index for babies; CSF, cerebrospinal fluid; IVH, intraventricular hemorrhage; PHH, post-hemorrhagic hydrocephalus. | | | |
