# Supplementary material for: Pro-inflammatory cerebrospinal fluid profile of neonates with intraventricular hemorrhage: clinical relevance and contrast with CNS infection
Source: Fluids Barriers CNS. 2024 Feb 21;21:17. doi: 10.1186/s12987-024-00512-0 (PMC10880312; doi:10.1186/s12987-024-00512-0)
Supplement: Supplementary file 2 — Additional file 2: Table S2. Bacterial and viral organisms identified in the cerebrospinal fluid of infants with meningitis. [file 12987_2024_512_MOESM2_ESM.docx]

| Additional file 2: Table S2. Bacterial and viral organisms identified in the cerebrospinal fluid of infants with meningitis. | |
| --- | --- |
| Organism or group | **N (%)** |
| Bacterial meningitis (n=20) |  |
| *Bacillus megaterium* | 2 (10) |
| *Group B streptococcus* | 2 (10) |
| *Coagulase-negative staphylococcus* | 7 (35) |
| *Cornebacterium* | 1 (5) |
| *E. coli* | 1 (5) |
| *Micrococcus* | 2 (10) |
| *S. capitis* | 1 (5) |
| *S. epidermidis* | 1 (5) |
| *S. hominis* | 1 (5) |
| *S. warneri* | 1 (5) |
| *Viridans streptococci* | 1 (5) |
|  |  |
| Viral meningitis (n=27) |  |
| *Enterovirus* | 21 (78) |
| *Herpes simplex virus* | 3 (11) |
| *Metapneumovirus* | 1 (4) |
| *Parechovirus* | 2 (7) |
