# Supplementary material for: Pro-inflammatory cerebrospinal fluid profile of neonates with intraventricular hemorrhage: clinical relevance and contrast with CNS infection
Source: Fluids Barriers CNS. 2024 Feb 21;21:17. doi: 10.1186/s12987-024-00512-0 (PMC10880312; doi:10.1186/s12987-024-00512-0)
Supplement: Supplementary file 3 — Additional file 3: Table S3. Cell counts by organism for bacterial and viral meningitis groups. [file 12987_2024_512_MOESM3_ESM.docx]

| Supplemental Table 3. Cell counts by organism for bacterial and viral meningitis groups. | | | | | | | | | |
| --- | --- | --- | --- | --- | --- | --- | --- | --- | --- |
| Organism or group | **N (%)** | **CSF** | | | | | | | |
|  |  | **Total cells (cells/µl)** | **WBC (cells/µL)** | **RBC (cells/µL)** | **Neutrophils (%)** | **Lymphocytes (%)** | **Monocytes (%)** | **Eosinophils (%)** | **Macrophages (%)** |
| Bacterial meningitis (n=20) |  |  |  |  |  |  |  |  |  |
| *Bacillus megaterium* | 2 (10) | 1993 ± 2586.6 | 15 ± 9.9 | 1978 ± 2576.7 | 19 ± 26.87 | 37 ± 1.41 | 37.5 ± 36.06 | 1 ± 1.41 | 0.5 ± 0.71 |
| *Group B streptococcus* | 2 (10) | 78.5 ± 21.92 | 42 ± 15.56 | 36 ± 37.48 | 17 ± 19.8 | 2 ± 1.41 | 46.5 ± 61.52 | 3.5 ± 3.54 | 0 |
| *Coagulase-negative staphylococcus* | 7 (35) | 2360.43 ± 4111.2 | 2.29 ± 1.98 | 2358.14 ± 4111.13 | 9.71 ± 13.2 | 35 ± 22.1 | 49.57 ± 28.01 | 0.57 ± 1.51 | 3.43 ± 7.46 |
| *Corynebacterium* | 1 (5) | 13 | 12 | 1 | 0 | 38 | 62 | 0 | 0 |
| *E. coli* | 1 (5) | 3492 | 1220 | 2275 | 637 | 7 | 29 | 1 | 0 |
| *Micrococcus* | 2 (10) | 13 ± 12.73 | 4 ± 4.24 | 9 ± 8.49 | 0 | 17 ± 9.9 | 74.5 ± 17.68 | 0 | 8.5 ± 7.78 |
| *S. capitis* | 1 (5) | 24 | 2 | 22 | 5 | 16 | 76 | 0 | 3 |
| *S. epidermidis* | 1 (5) | 70 | 1 | 69 | 17 | 44 | 39 | 0 | 0 |
| *S. hominis* | 1 (5) | 2 | 1 | 1 | 6 | 6 | 55 | 0 | 33 |
| *S. warneri* | 1 (5) | 1866 | 1050 | 816 | 60 | 10 | 26 | 1 | 0 |
| *Viridans streptococci* | 1 (5) | 0 | 0 | 0 | 0 | 0 | 0 | 0 | 0 |
| Viral meningitis (n=27) |  |  |  |  |  |  |  |  |  |
| *Enterovirus* | 21 (78) | 1315.14 ± 3061.33 | 79.05 ± 213.41 | 1236.1 ± 3083.42 | 28.24 ± 33.09 | 17.9 ± 10.32 | 46.05 ± 31.4 | 0 | 5.1 ± 12.47 |
| *Herpes simplex virus* | 3 (11) | 3714.33 ± 5239.77 | 124.67 ± 173.7 | 3589.67 ± 5328.47 | 22.33 ± 38.68 | 36.33 ± 21.5 | 34.67 ± 29.14 | 0 | 2.67 ± 3.06 |
| *Metapneumovirus* | 1 (4) | 2150 | 0 | 2150 | 13 | 65 | 22 | 0 | 0 |
| *Parechovirus* | 2 (7) | 964.5 ± 1358.35 | 14 ± 15.56 | 950.5 ± 1342.8 | 0 | 44 ± 32.53 | 56 ± 32.53 | 0 | 0 |
| Data shown as mean ± standard deviation. Abbreviations: CSF, cerebrospinal fluid; RBC, red blood cell; WBC, white blood cell. | | | | | | | | | |
